# Case Report: Iatrogenic Bowel Perforation Following Dental Procedure

**DOI:** 10.21980/J8CD38

**Published:** 2025-07-31

**Authors:** Claire DeLong, Frederick Fiesseler

**Affiliations:** * Morristown Medical Center, Department of Emergency Medicine, Morristown, NJ

## Abstract

Abdominal pain is a common complaint in emergency medicine, with a vast differential diagnosis. We report a case of a 42-year-old male presenting with two days of progressively worsening left lower quadrant (LLQ) pain. A CT scan of the abdomen demonstrated a small metallic foreign body. The patient had not ingested any grilled food, since this was the speculated etiology, given the concern for potentially ingesting a wire grill brush piece. Considering the significant pain and associated computer tomography (CT) findings, the patient was taken to the operating room. A 2 cm piece of metal wire was found perforating his small intestines. The patient remembered a recent visit to his orthodontist which was uneventful. Complications from orthodontic procedures are rare and typically benign. We report an unusual case of an iatrogenic small bowel perforation from an archwire. The patient had a full recovery.

**Topics:**

Orthodontist, bowel perforation, iatrogenic, archwire.

**Figure:**
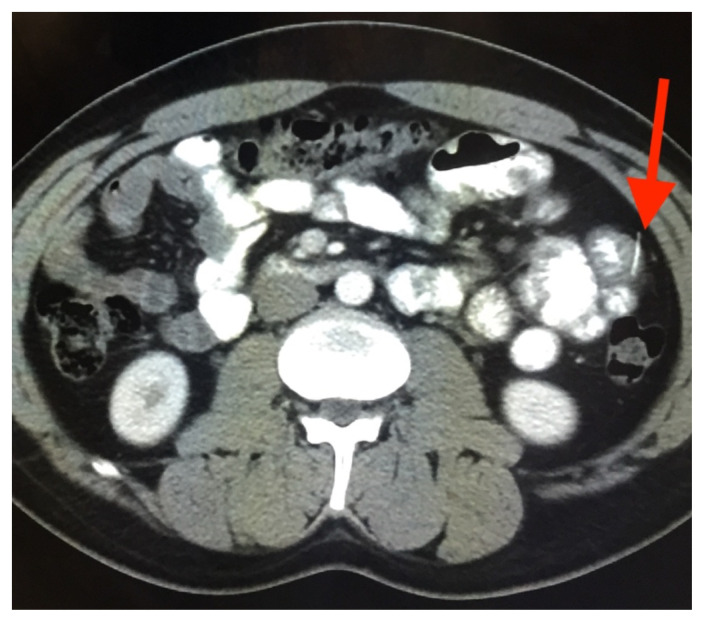


**Figure:**
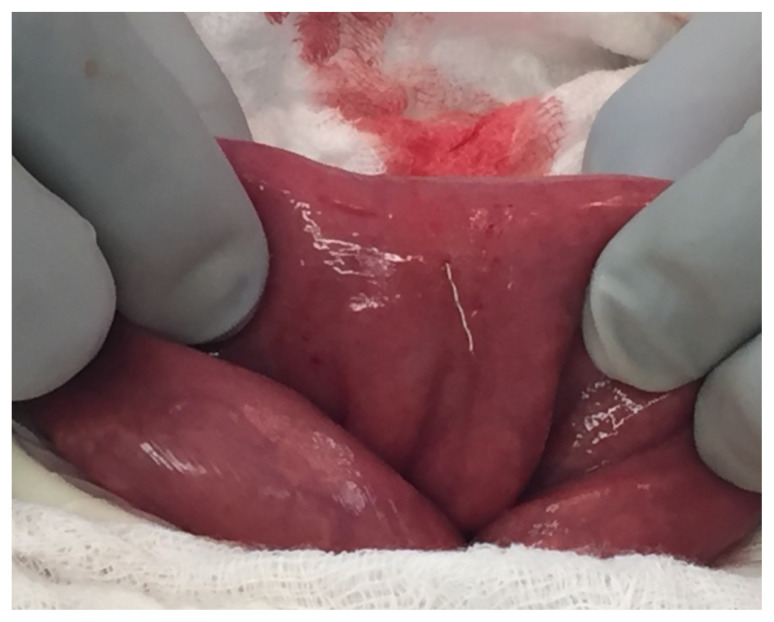


## Brief introduction

Abdominal pain is one of the most frequently encountered presenting symptoms in emergency medicine, with a frequency of 7–10% of all visits.^1^ There is a broad differential diagnosis, with the spectrum being from benign to life-threatening etiologies.^1^ A thorough history and physical exam can help delineate the cause of symptoms. When this does not suffice, ancillary diagnostics including labs and radiological imaging, may be warranted. Orthodontic procedures are common, with low complication rates of only 1.8% for patients with brackets and bands.^2^ Despite a low complication rate, recent procedures performed is an important aspect in a patient’s history.^2^ Potential complications of dental and orthodontic procedures are essential for Emergency Physicians to know because these rare complications can lead to significant infection, laceration, pain, and even airway compromise; thus, prompt recognition is essential.

## Presenting concerns and clinical findings

A 42-year-old male, with no relevant past medical or family history, presented to the emergency department with LLQ pain, which had progressively worsened over the last day. The patient stated the pain worsened with palpation of his abdomen but denied any remitting factors. He reported no changes in his bowel or bladder function but did admit to decreased appetite. He did not ingest grilled food recently, so the suspicion of ingested brush wire was low. He reported having an orthodontic procedure one week prior. The patient continued to have localized tenderness in the LLQ with associated guarding but without peritoneal signs on re-exam. Blood work demonstrated a mild leukocytosis of 11.5×10^3^/μL. All other bloodwork was within normal limits. The patient underwent abdominal CT with contrast.

## Significant findings

The patient’s abdominal CT demonstrated a metallic foreign body in the left side of the abdomen within the small bowel, without surrounding induration or abscess. Radiology questioned whether the metallic foreign object perforated the bowel. Seen in the cross-sectional CT image, there is a hyperdense linear structure transversing the small intestinal wall, given that a portion of the structure was located outside of the lumen of the bowel.

## Patient course

Surgery was consulted regarding the metallic foreign body found on CT with possible perforation, and he was taken to the operating room (OR) for diagnostic laparotomy. In the OR, the patient was found to have a 2 cm wire perforating his small bowel without evidence of further intra-abdominal injury. The wire was removed, and a suture was placed over the site. During the hospital stay, he remembered an incident that occurred at his orthodontist in which the orthodontist appeared concerned after losing a piece of material during the patient’s archwire replacement. The patient again denied any other possible sources of this foreign body. Two days after surgery the patient was discharged home without further incident. The patient had no long-term sequela.

## Discussion

Complications from oral surgery/dental procedures are often of little clinical significance and include pain, bleeding, bruising, swelling, and dry socket. Some of the more serious complications include nerve injury (eg, inferior alveolar and lingual nerves), allergic reaction, infection, and foreign body aspiration/ingestion. Aspiration or ingestion are uncommonly reported events.^3^ There is a limitation of the literature published in this area about retained dental hardware, and thus an often-missed piece of the history in the emergency setting. Emergency physicians must be thorough about asking about prior procedures and surgical history. Frequently, advanced radiological exams are required to determine the location of the foreign body due to its small size. Most ingestions are reported to be prostheses or crowns.^4^ It is imperative to determine that the foreign body is not retained within the pulmonary system (~13% of the time) as these will require surgical extraction.^5^ The remaining 87% will enter the alimentary tract and approximately 90% of those will pass through uneventfully.^4^,^5^ The size, sharpness, and shape will often influence the management and outcomes. We presented a rare case of iatrogenic foreign body ingestion resulting in bowel perforation.

Recently, a number of cases have been reported regarding grill brush bristle ingestion.^6^–^10^ Grill bristles are of a similar size and shape as an archwire. Archwires have additionally been reported in the upper aerodigestive tract, but the data is less robust.^11^ Grill bristle ingestion seems to predominantly become lodged in the upper aerodigestive tract, followed by the abdomen. Localizing the foreign body can be difficult utilizing radiographs and laryngoscopy, often requiring CT which has a 92.8% sensitivity rate; when suspicion is high and initial imaging is negative, patients require observation for continued assessments.^9^–^10^ Early surgical consultation is recommended when the suspicion for injury is high. Abdominal complications of inadvertent bristle ingestion included: ureteral obstruction, inflammation suspicious for appendicitis, sepsis leading to death, and pancreatic injury, as well as abscess of the extraluminal duodenum, abdominal wall and peritoneum.^10^ Reports of abdominal complications rarely occur with dental foreign bodies.^12^–^13^ It is speculated that grill brush bristles ingestion would have a similar complication response.

Litigation exists on cases of missed wire bristle retention that have led to adverse patient outcomes and jury verdicts awarded to the plaintiff. In 2017, the American Journal of Orthodontics and Dentofacial Orthopedics published a case of an EM physician who was sued after a patient was found to have an “errant piece of archwire” below the diaphragm on x-ray, following a dental procedure. The patient was discharged home after stating that due to the location, the foreign body would be passed in the stool with conservative management, and she was given ED return precautions. Unfortunately, she returned one day later with a bowel perforation, leading to sepsis and requiring multiple surgeries.^14^ It was thought that the plaintiff should have been notified about the possibility of endoscopic removal on initial presentation.^14^ The verdict was found in her favor. Ingesting a foreign body is an ever-present risk during dental procedures, with prevention being the primary goal. When a complication occurs, the provider has the duty to disclose the complication to the patient. When cases like this occur, a CT of the abdomen and pelvis is warranted to exclude perforation, and shared decision-making should occur regarding their care.

While complications such as this are rare, and literature on best practices is sparse, it demonstrates the importance of asking about recent procedures and surgeries. If there is a high clinical suspicion of injury, early surgical consultation should be considered, given the diagnostic challenges that may be present.

## Supplementary Information






