# Supplementary figures and images for: Case Report: Iatrogenic Bowel Perforation Following Dental Procedure
Source: J Educ Teach Emerg Med. 2025 Jul 31;10(3):V5–7. doi: 10.21980/J8CD38 (PMC12320992; doi:10.21980/J8CD38)

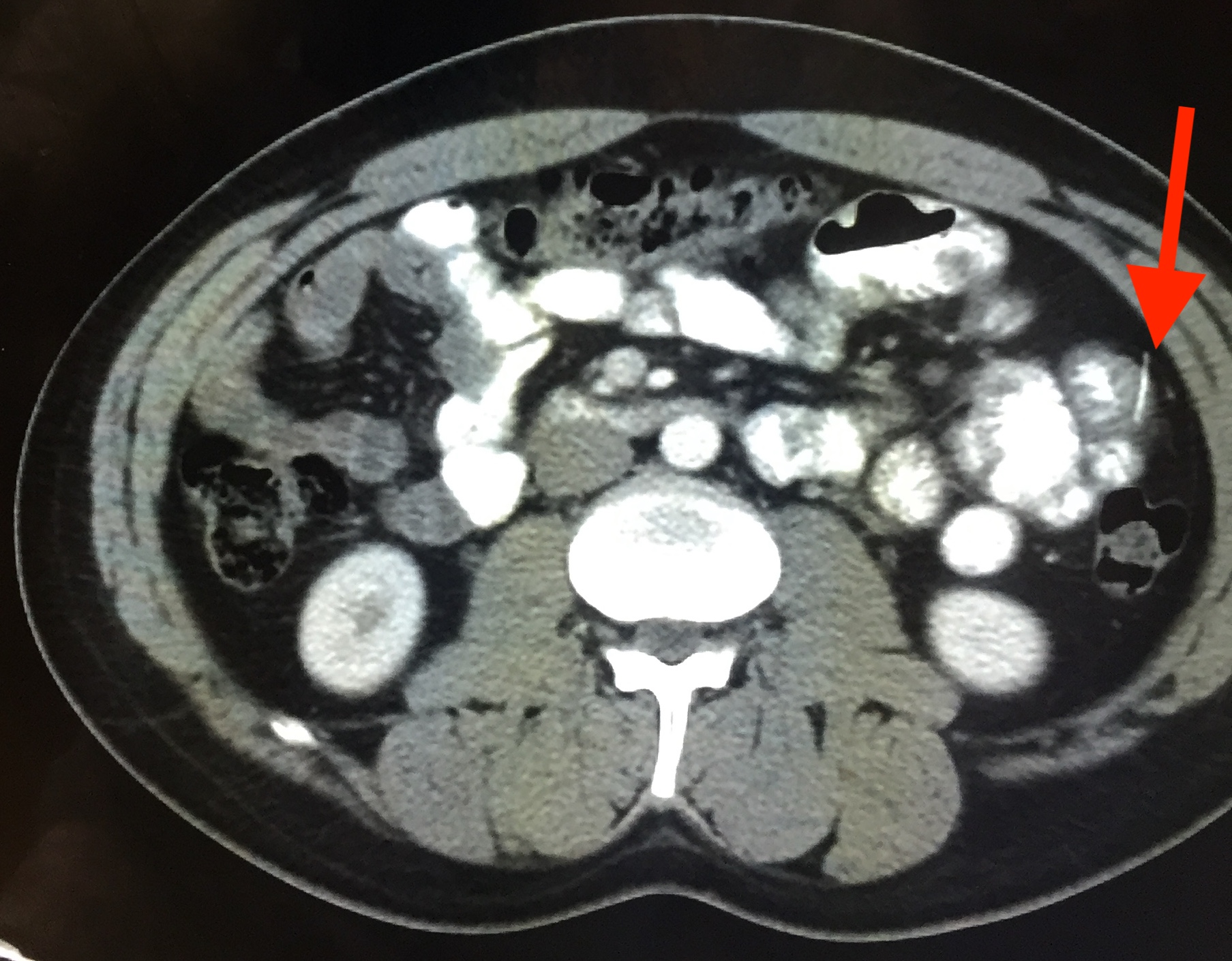

Supplement: Supplementary file 1 [file 10-3-V5-supp1.jpg]

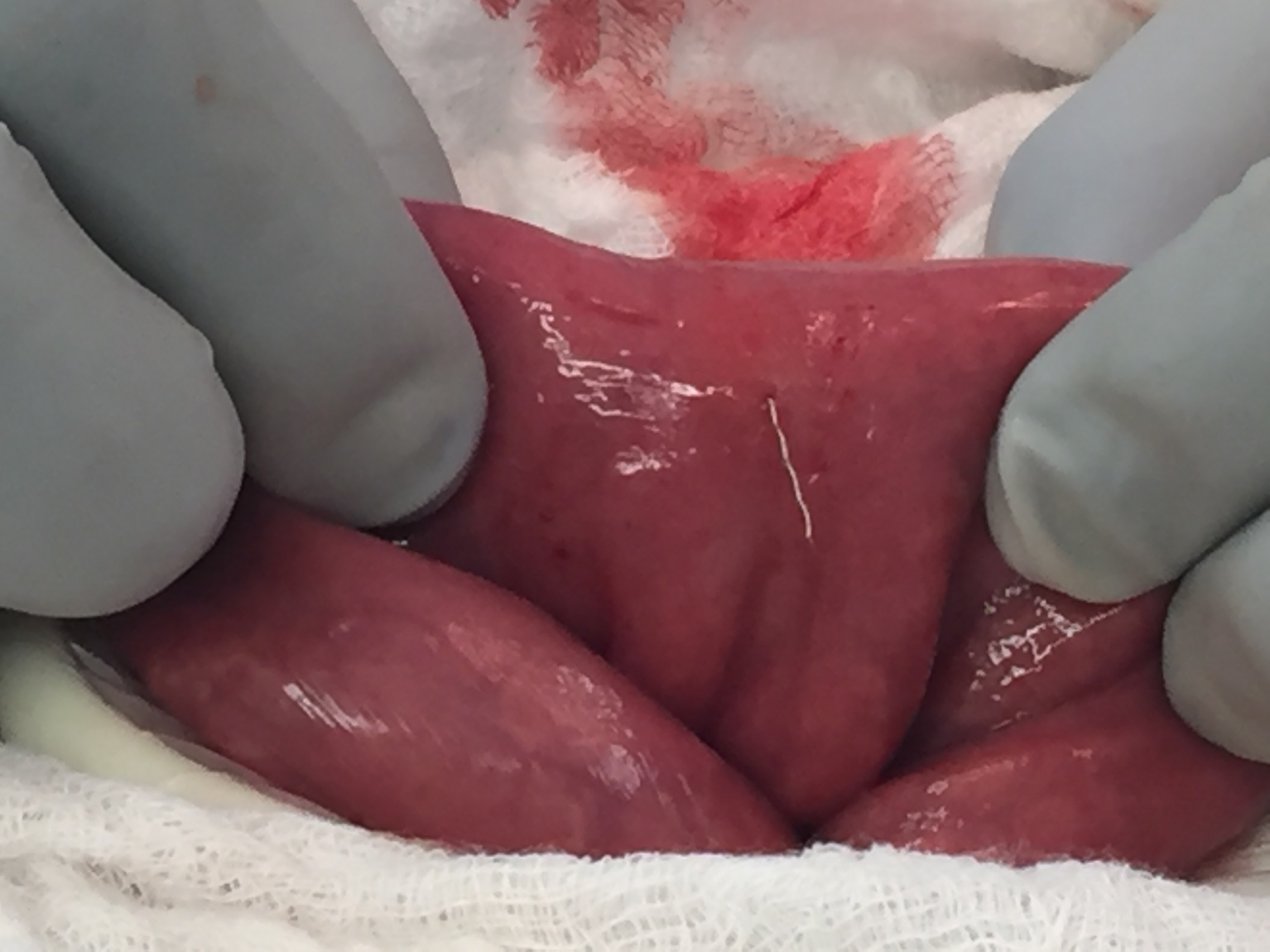

Supplement: Supplementary file 2 [file 10-3-V5-supp2.jpg]

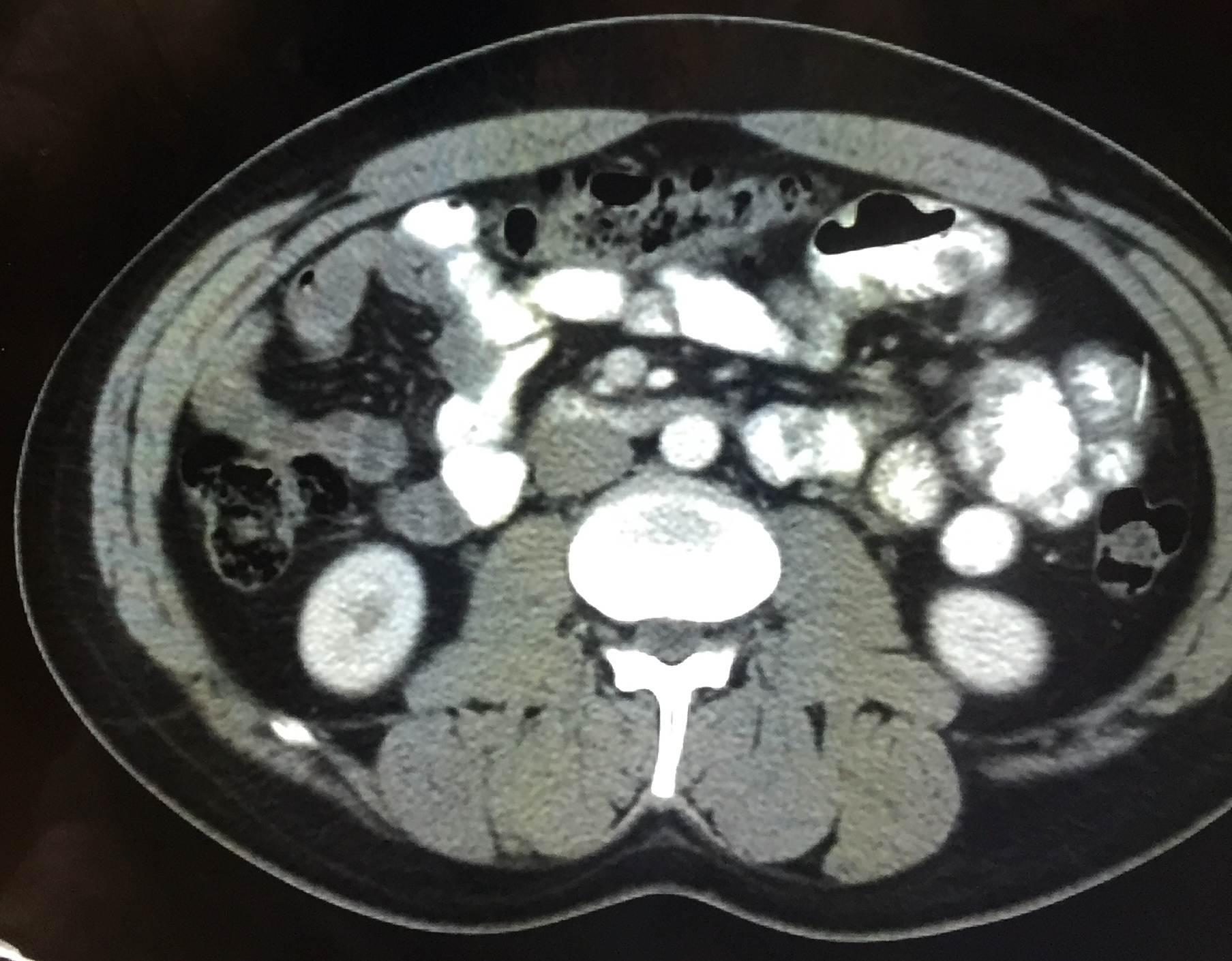

Supplement: Supplementary file 3 [file 10-3-V5-supp3.jpg]
